# CRIF1 overexpression facilitates tumor growth and metastasis through inducing ROS/NFκB pathway in hepatocellular carcinoma

**DOI:** 10.1038/s41419-020-2528-7

**Published:** 2020-05-07

**Authors:** Hulin Chang, Juntang Li, Kai Qu, Yong Wan, Sinan Liu, Wei Zheng, Zhiyong Zhang, Chang Liu

**Affiliations:** 1 Department of Hepatobiliary Surgery, The First Affiliated Hospital, College of Medicine, Xi’an Jiaotong University, Xi’an, 710061 Shaanxi China; 2 Department of Hepatobiliary Surgery, The Third Affiliated Hospital, College of Medicine, Xi’an Jiaotong University, Xi’an, 710068 Shaanxi China; 3 Centre of Inflammation and Cancer Research, Anal-Colorectal Surgery Institute of PLA, Luoyang, 471031 Henan China; 4 Department of Pathology, 150th Central Hospital of PLA, Luoyang, 471031 Henan China

**Keywords:** Liver cancer, Liver cancer

## Abstract

CR6-interacting factor 1 (Crif1) is a mitochondrial protein which is required for the assembly of oxidative phosphorylation (OXPHOS) complexes. Our bioinformatics analysis based on Cancer Genome Atlas (TCGA) database revealed an aberrant overexpression of CRIF1 in hepatocellular carcinoma (HCC). However, the clinical significance and biological functions of CRIF1 are still unclear in this malignancy. Here, we report that CRIF1 is frequently overexpressed in HCC cells mainly due to the downregulation of miR-497-5p, which is associated with poor prognosis of patients with HCC. CRIF1-promoted HCC growth and metastasis by suppressing cell apoptosis and inducing cell cycle progression and epithelial to mesenchymal transition (EMT). Mechanistically, increased mitochondrial ROS production and consequently activation of the NFκB signaling pathway was found to be involved in the promotion of growth and metastasis by CRIF1 in HCC cells. In summary, CRIF1 plays an oncogenic role in HCC progression through activating ROS/NFKB pathway, implying CRIF1 as a potential prognostic factor and therapeutic target in HCC.

## Introduction

Liver cancer, primarily encompassing hepatocellular carcinoma (HCC), is one of the commonest causes of cancer death worldwide^1^. The prognosis of HCC patients remains dismal despite the advances in surgical and adjuvant systemic treatment have been achieved^2^. Mitochondria are critical organelles possess numerous bioenergetic and biosynthetic functions in mammalian cells^3^. In addition, mitochondria are well appreciated as a major source of reactive oxygen species (ROS)^4,5^, which lead to the activation of several key oncogenic signaling pathways involved in tumor growth and metastasis^6^. Although cumulative evidence has revealed the close links between mitochondrial dysfunction and tumor progression in various types of human cancers^7–9^, the novel molecular mechanism underlying mitochondrial dysfunction-mediated tumorigenesis remains largely unknown.

CR6-interacting factor 1 (Crif1) has been characterized as a novel factor involved in the assembly of oxidative phosphorylation (OXPHOS) complexes in mitochondrial through regulation of the synthesis and insertion of OXPHOS polypeptides into the inner membrane of mitochondrial in mammals^10^. However, as a novel regulator of mitochondrial OXPHOS, the expression and biological effects of CRIF1 in cancer development and progression are still unknown, especially in HCC.

Our bioinformatics analysis based on the data from The Cancer Genome Atlas (TCGA) revealed an aberrant overexpression of CRIF1 in HCC, indicating that CRIF1 may play a role in the progression of HCC. We conducted the first study on the role of CRIF1 in HCC focused on its clinical significance, biological effects, and the underlying mechanisms in this malignancy.

## Results

### CRIF1 was frequently overexpressed in HCC and contributed to worse prognosis

**Fig. 1:**
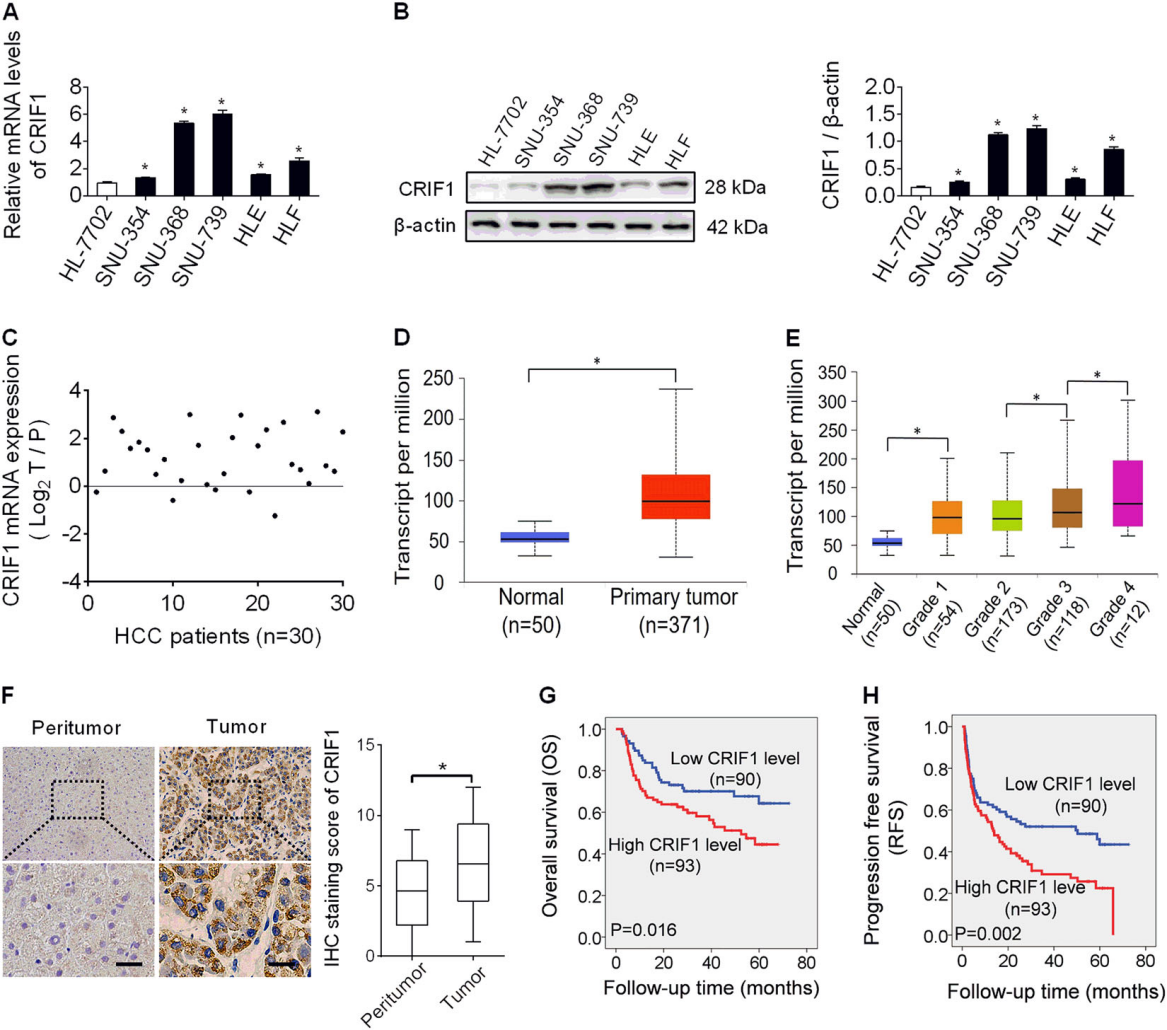
CRIF1 was frequently overexpressed in HCC and contributes to worse prognosis. **a** Quantitative real-time PCR (qRT-PCR) analysis for mRNA expression levels of CRIF1 in five human HCC cell lines (SNU-354, SNU-368, SNU-739, HLE, and HLF) and one normal human hepatic cell line HL-7702. **b** Western blot analysis for protein expression levels of CRIF1 in five human HCC cell lines (SNU-354, SNU-368, SNU-739, HLE, and HLF) and one normal human hepatic cell line HL-7702. **c** Quantitative RT-PCR analysis for mRNA expression levels of CRIF1 in paired tumor and peritumor tissues from 30 HCC patients. (T tumor, P peritumor). **d**, **e** UALCAN (a portal for facilitating tumor subgroup gene expression and survival analyses) websites-based bioinformatic analysis of CRIF1 expression in HCC. **f** Immunohistochemical staining for expression levels of CRIF1 in paired tumor and peritumor tissues from 183 HCC patients. Left panel: representative immunohistochemical (IHC) staining images of CRIF1 in paired tumor and peritumor tissues of HCC. Scale bar, 50 μm. Right panel: IHC staining scores of CRIF1 in paired tumor and peritumor tissues of HCC. **g**, **h** Overall survival (OS) and recurrence-free survival (RFS) were evaluated by the Kaplan–Meier survival curves and the log-rank test in tumor tissues from 183 HCC patients according to CRIF1 expression levels. Data are expressed as mean ± SEM from three independent experiments. *p < 0.05.

We firstly evaluated the expression of CRIF1 in HCC cell lines and human tissue samples. CRIF1 expression was dramatically enhanced in all five HCC cell lines (SNU-354, SNU-368, SNU-739, HLE, and HLF) compared with normal human hepatocytes HL-7702 at both mRNA and protein levels (Fig. 1a, b). In concordance with the results from cell lines, CRIF1 was also significantly upregulated in 30 primary HCC tissues when compared with their adjacent normal tissues by the qRT-PCR analysis (Fig. 1c). To provide further evidence, the mRNA expression of CRIF1 in TCGA database was further analyzed based on the UALCAN websites, which is a portal for facilitating tumor subgroup gene expression and survival analyses^11^. As shown in Fig. 1d, CRIF1 expression is significantly higher in tumor tissues of HCC (*n* = 371) when compared with normal samples (*n* = 50) (*P* < 0.0001). In addition, CRIF1 expression is also higher in high grade tumors than that in low grade tumors (Fig. 1e). To explore the association between CRIF1 expression and the clinicopathological features and clinical outcomes of HCC patients, CRIF1 expression was evaluated by the immunohistochemistry (IHC) analysis in tumor tissues from 183 patients with HCC. Representative IHC staining images and intensity scores (Fig. 1f) indicated that CRIF1 expression is significantly higher in HCC tissues compared with adjacent non-tumor tissues. There was no significant correlation between the expression of CRIF1 and age, sex, virus infection, and serum alpha fetoprotein, while higher CRIF1 expression level was significantly associated with larger tumor size and higher TNM stage (Table S1). Kaplan–Meier analysis indicated that high expression of CRIF1 was significantly associated with poor overall survival (OS) and recurrence-free survival (RFS) in patients with HCC (Fig. 1g, h).

### CRIF1-promoted HCC growth by inducing G1–S cell cycle transition and suppressing cell apoptosis/necrosis

**Fig. 2:**
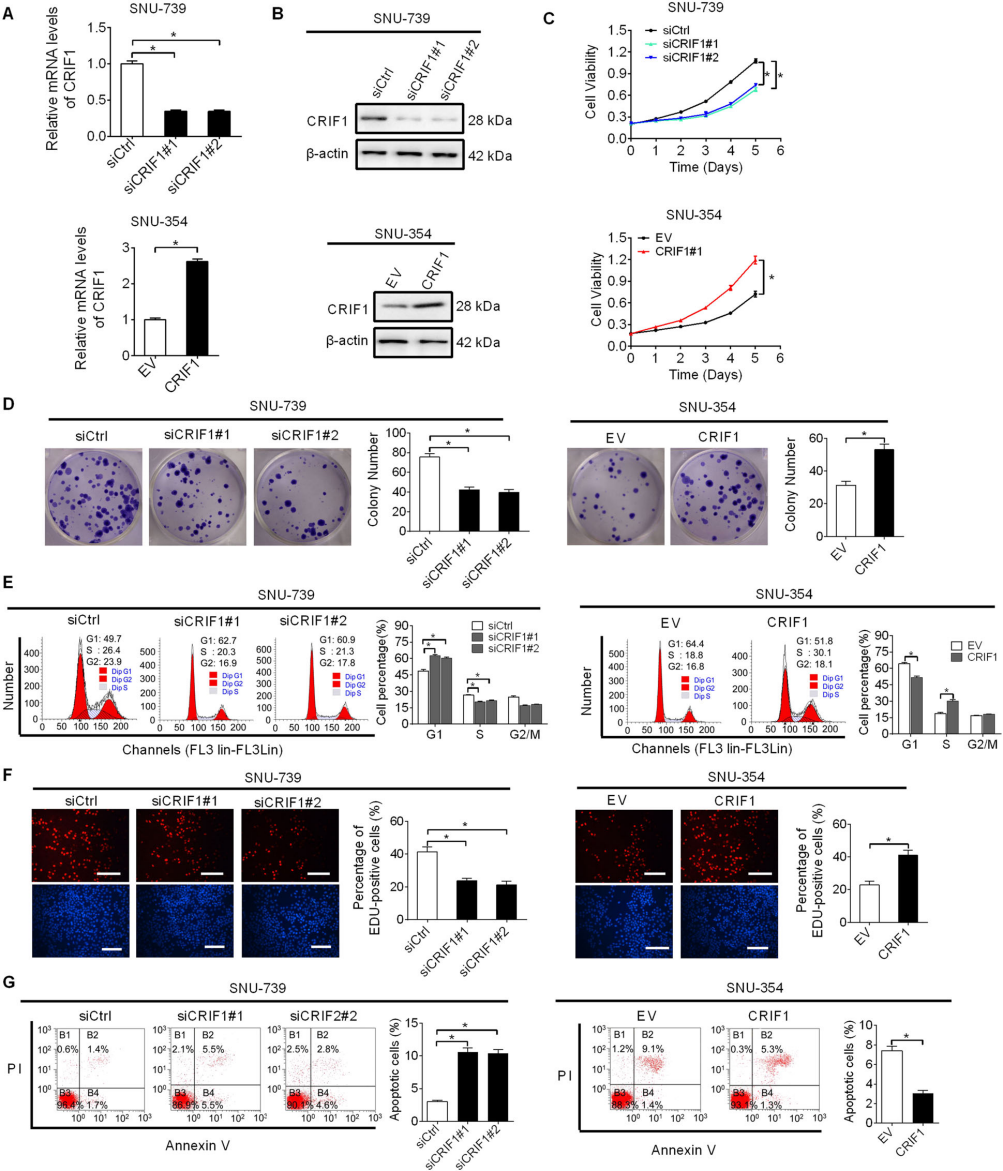
CRIF1-promoted HCC growth by inducing G1–S cell cycle transition and suppressing cell apoptosis. **a**, **b** The efficiency of knockdown and overexpression of CRIF1 were verified by qRT-PCR and western blot analysis in SNU-739 and SNU-354 cells with indicated treatment for 48 h (siCRIF1, siRNA against CRIF1; siCtrl, control siRNA; EV, empty vector; CRIF1, expression vector encoding CRIF1). **c** MTS cell viability assay in SNU-739 and SNU-354 cells with treatment for 48 h as indicated. **d** Colony formation assay in SNU-739 and SNU-354 cells with treatment for 48 h as indicated. **e** Flow cytometry analysis for cell cycle distribution in SNU-739 and SNU-354 cells with treatment for 48 h as indicated. **f** EdU incorporation assay for track proliferating cells (red indicates BrdU-incorporated cells; blue indicates nuclei staining with Hoechst) was performed in SNU-739 and SNU-354 cells with treatment for 48 h as indicated. Scale bars, 50 μm. **g** Flow cytometry analysis for cell apoptosis/necrosis in SNU-739 and SNU-354 cells with treatment for 48 h as indicated. Data are expressed as mean ± SEM from three independent experiments. *p < 0.05.

To determine the biological functions of CRIF1 in HCC, we examined the effects of CRIF1 on the growth characteristics of HCC cells with MTS and colony formation assays. CRIF1 expression was knocked down in SNU-739 cells with relative high CRIF1 expression and overexpressed in SNU-368 cells with relative low CRIF1 expression. The efficiency of knockdown or overexpression of CRIF1 was verified by RT-PCR (Fig. 2a) and western blot (Fig. 2b) analysis. As shown in Fig. 2c, d, knockdown of CRIF1 significantly attenuated cell growth and inhibited clonogenicity in SNU-739 cells, while overexpression of CRIF1 significantly accelerated cell growth and increased clonogenicity in SNU-354 cells.

Increased HCC growth could be due to accelerated cell cycle progression or decreased apoptosis, or both. To explore the mechanism by which CRIF1-promoted HCC cell growth, the effects of CRIF1 on cell cycle distribution and apoptosis were determined by flow cytometry. CRIF1 knockdown resulted in a significant increase of cells in G1 phase and a concomitantly significant decrease of cells in S phase in SNU-739 cells, while CRF1 overexpression exhibited an opposite effect in SNU-354 cells (Fig. 2e). EdU (5-ethynyl-2′-deoxyuridine) incorporation assay also showed significantly fewer proliferating cells in SNU-739 cells with CRIF1 knocked down, while significantly more proliferating cells in SNU-354 cells with CRIF1 overexpressed when compared with their control cells (Fig. 2f). Apoptosis analysis showed CRIF1 knockdown significantly increased the percentage of apoptotic/necrotic cells in SNU-739 cells, while CRF1 overexpression remarkably reduced the percentage of apoptotic/necrotic cells in SNU-354 cells (Fig. 2g). These results collectively indicate that CRIF1 promote HCC growth mainly through inducing G1–S cell cycle transition and suppressing cell apoptosis.

### CRIF1 enhanced invasion and migration abilities of HCC cells through induction of epithelial–mesenchymal transition (EMT)

**Fig. 3:**
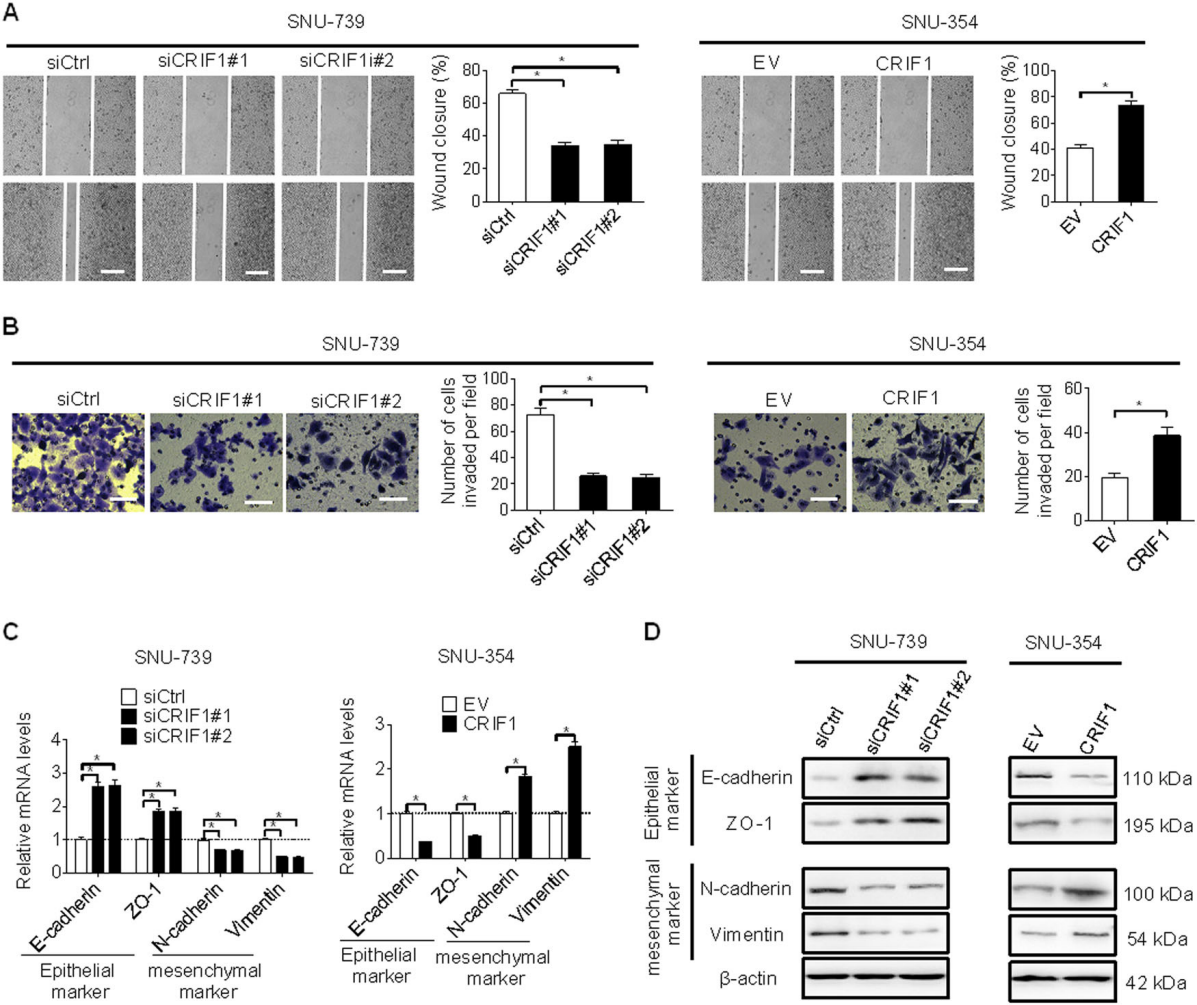
CRIF1 enhanced invasion and migration abilities of HCC cells through induction of epithelial–mesenchymal transition (EMT). **a** Cell migration ability was determined by wound healing assay in SNU-739 and SNU-354 cells with indicated treatment for 48 h (siCRIF1, siRNA against CRIF1; siCtrl, control siRNA; EV, empty vector; CRIF1, expression vector encoding CRIF1). Vertical line indicate boundary of cell wound, horizontal lines indicate scale bars, 50 μm. **b** Cell invasion ability was determined by the transwell matrigel invasion assay in SNU-739 and SNU-354 cells with indicated treatment for 48 h (Scale bars, 20 μm). **c**, **d** Quantitative RT-PCR and western blot analysis for expression levels of EMT markers (E-cadherin, ZO-1, N-cadherin, and vimentin) in SNU-739 and SNU-354 cells with indicated treatment for 48 h. Data are expressed as mean ± SEM from three independent experiments. *p < 0.05.

We also investigated the effect of CRIF1 on the metastasis of HCC with wound healing and transwell invasion assays. Knockdown of CRIF1 dramatically reduced the migratory and invasive abilities of SNU-739 cells, while overexpression of CRIF1 significantly increased the migratory and invasive abilities of SNU-354 cells (Fig. 3a, b).

It has been well-established that EMT plays a critical role in promoting tumor metastasis^12^. To determine whether EMT plays a role in CRIF1-promoted HCC migration and invasion, the expression levels of EMT markers were determined by qRT-PCR and western blot analysis. As shown in Fig. 3c, d, CRIF1 knockdown significantly increased the expression levels of epithelial markers (E-cadherin and ZO-1), while decreased the expression levels of mesenchymal markers (N-cadherin and vimentin) in SNU-739 cells. In contrast, CRIF1 overexpression exhibited an opposite effect in SNU-354 cells. These results suggest that CRIF1 promoted the migration and invasive abilities of HCC cells mainly through inducing EMT.

### CRIF1 knockdown attenuated the growth and metastasis of HCC in vivo

**Fig. 4:**
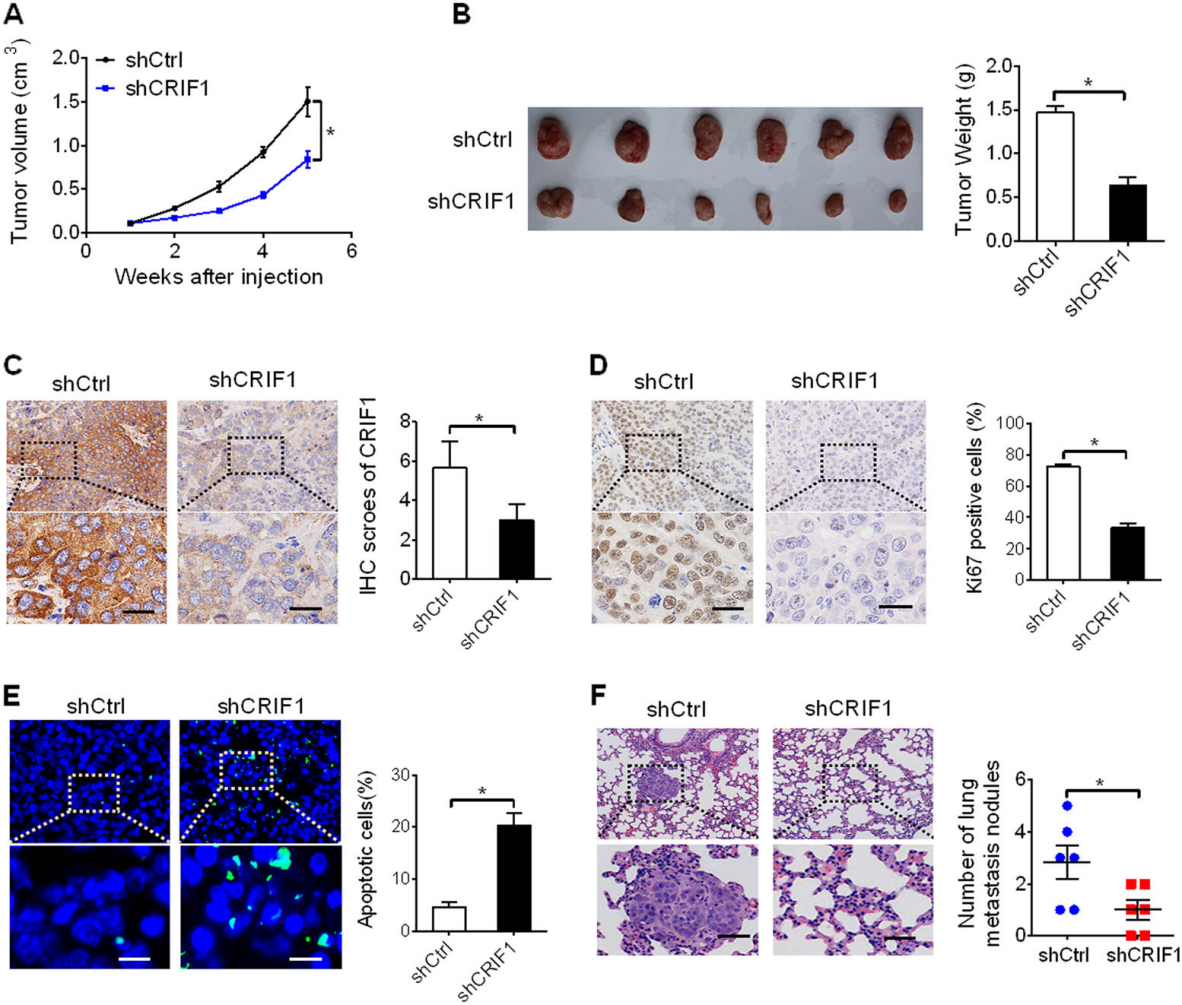
CRIF1 knockdown attenuated the growth and metastasis of HCC in vivo. **a** Tumor growth curves of subcutaneous xenografts in nude mice established from SNU-368 cells with stable CRIF1-knockdown or control cells. (shCRIF1, shRNA expression vector against CRIF1; shCtrl, control shRNA). **b** Tumors were dissected from the mice and the wet weight of tumors in shCRIF1 and shCtrl groups was compared. **c** The expression of CRIF1 in subcutaneous xenografts was confirmed by immunohistochemistry (IHC) analysis in subcutaneous xenografts from shCRIF1 and shCtrl groups (left panel: representative IHC staining images. Scale bar, 50 μm; right panel: IHC staining intensity). Scale bar, 50 μm. **d** IHC staining for Ki-67 in subcutaneous xenografts from shCRIF1 and shCtrl groups. Scale bar, 50 μm. **e** Representative TUNEL staining and the percentages of apoptotic cells in subcutaneous xenografts from shCRIF1 and shCtrl groups were shown. Scale bars, 20 μm. **f** Representative lung metastatic foci and the incidences of lung metastases in each group were shown. Scale bars, 50 μm. Data are expressed as mean ± SEM from three independent experiments. *p < 0.05.

To assess the role of CRIF1 in tumorigenesis of HCC in vivo, we performed a xenograft model assay through subcutaneously injecting stable CRIF1-knockdown or control SNU-739 cells into the flank of nude mice. As shown in Fig. 4a, the tumor growth rate was significantly slower in shCRIF1-transfected nude mice than in control (shCtrl) mice. The net weight of tumors formed by CRIF1-knockdown cells was also significantly decreased compared with the control (shCtrl) group at the termination of the experiment (Fig. 4b). IHC analysis indicated that the expression of CRIF1 was greatly decreased in xenograft tumors from the shCRIF1 group when compared with the control (shCtrl) group (Fig. 4c), indicating that the tumor growth suppressive effect was exerted by knockdown of CRIF1. Ki-67 and TUNEL staining assays further demonstrated significantly fewer proliferating and more apoptotic cells in tumor tissues from shCRIF1 xenografts compared with the control (shCtrl) (Fig. 4d, e).

To further assess the function of CRIF1 on metastasis of HCC cells in vivo, CRIF1-knockdown and control SNU-739 cells were intravenously injected into the nude mice. After 40 days of injection, the mice were killed and their lungs were collected. As shown in Fig. 4f, the number of metastasis nodules in the lungs from shCRIF1 group was significantly decreased when compared with those from the control (shCtrl) group.

### CRIF1-promoted HCC growth and metastasis through activation of ROS/NFκB signaling

**Fig. 5:**
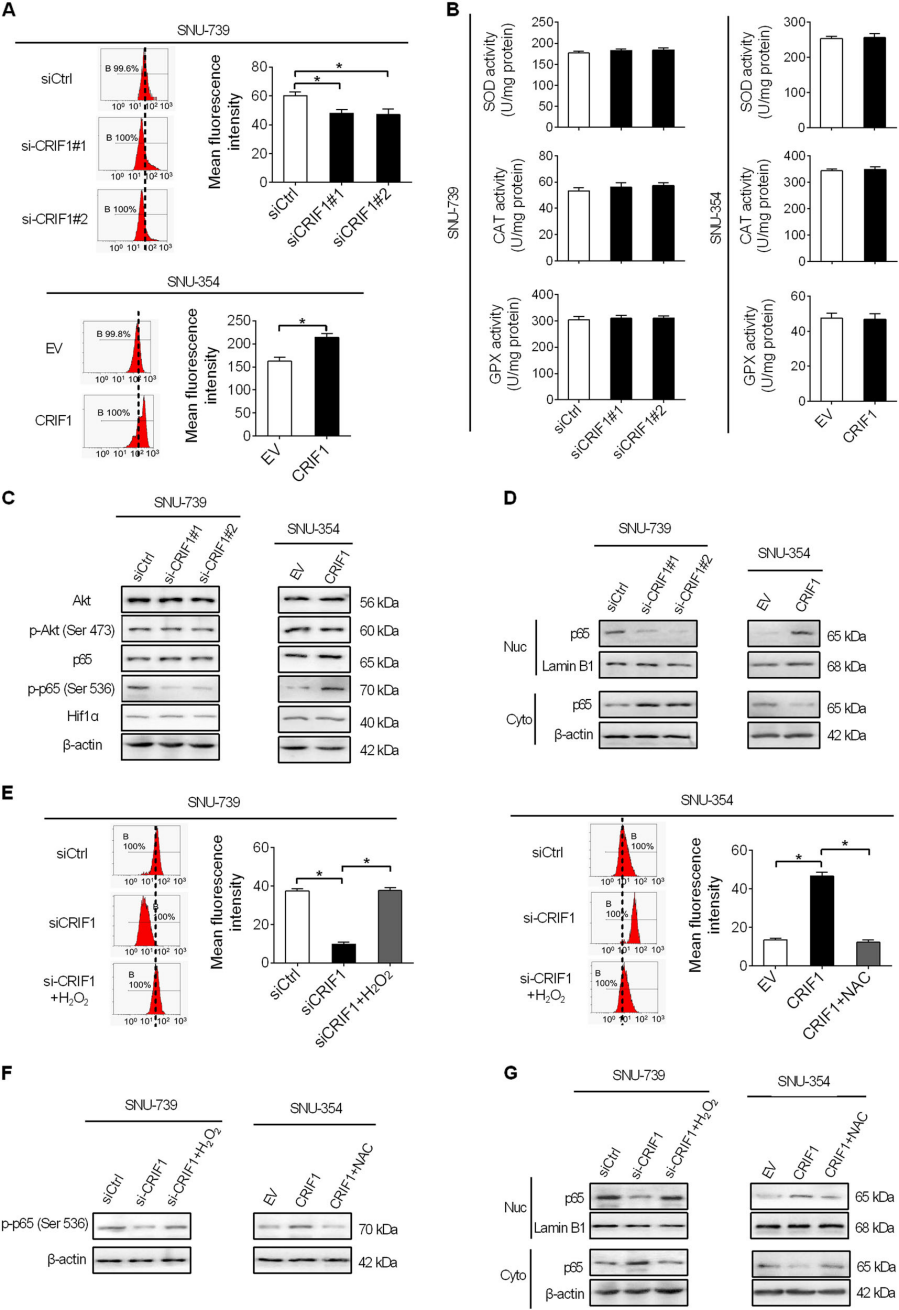
CRIF1 activated ROS/NFκB signaling in HCC cells. **a** Detection of intracellular ROS levels by flow cytometry analysis in SNU-739 and SNU-354 cells with treatment as indicated (siCRIF1, siRNA against CRIF1; siCtrl, control siRNA; EV, empty vector; CRIF1, expression vector encoding CRIF1). **b** Detection of activities of SOD, CAT, and GPX in SNU-739 and SNU-354 cells with treatment as indicated. **c** Western blot analyses for levels of Akt, p-Akt (Ser473), p65, p-p65 (Ser536), and HIF1a in SNU-739 and SNU-354 cells with treatment as indicated. **d** Western blot analyses for nuclear and cytosolic levels of p65 in NU-739 and SNU-354 cells with treatment as indicated. **e** Intracellular ROS levels analyzed by flow cytometry in SNU-739 and SNU-354 cells treated as indicated. Cells were treated with 100 mM H_2_O_2_ or 20 mM NAC for 12 h. **f**, **g** Western blot analyses for total cellular levels of p-p65 (Ser536) and nuclear and cytosolic levels of p65 in SNU-739 and SNU-354 cells treated as indicated. Cells were treated with 100 mM H_2_O_2_ or 20 mM NAC for 12 h.

CRIF1 is a mitochondrial protein required for the translation of mtDNA-encoded respiratory subunits^13^. Previous studies have demonstrated that knockdown of CRIF1 induced mitochondrial dysfunction and subsequent ROS production^14,15^. Considering that ROS plays a critical role in the regulation of tumor growth and metastasis by activating several key oncogenic signaling pathways, such as AKT, NFκB, and Hif-1α^16^, we hypothesized that increased ROS level and activities of its downstream oncogenic signaling pathways may be involved in CRIF1-promoted HCC growth and metastasis. As shown in Fig. 5a, knockdown of CRIF1 in SNU-739 cells significantly reduced ROS levels, while forced CRIF1 expression in SNU-354 cells significantly elevated ROS levels, as measured by the flow cytometry. A significant positive correlation between CRIF1 expressions and ROS levels also has been observed in human HCC and normal hepatic cell lines (Fig. S1). Cellular ROS level is mainly determined by the balance between ROS generation and the antioxidant system. To explore the potential contribution of antioxidant system in CRIF1-increased ROS level in HCC, the effect of CRIF1 on the activities of three major antioxidant enzymes^17,18^, including superoxide dismutase (SOD), catalase (CAT), and glutathione peroxidase (GPX) were determined. As shown in Fig. 5b, both CRIF1 knockdown and overexpression did not notably affect the activities of SOD, CAT, and GPX, suggesting that CRIF1 increased cellular ROS level through increased production but not decreased antioxidant capacity. In addition, we found that knockdown of CRIF1 suppressed the activation of NF-κB signaling, while did not notably affected the activations of AKT and Hif-1α. In contrast, forced CRIF1 expression activated NF-κB signaling (Fig. 5c, d). To further test whether increased ROS level contributed to the activation NF-κB, H_2_O_2_ or NAC (an ROS scavenger) was treated to change cellular ROS levels in HCC cells. As shown in Fig. 5e, H_2_O_2_ treatment significantly increased ROS in SNU-739 cells with CRIF1 knocked down, while NAC treatment markedly decreased ROS level in SNU-354 cells with CRIF1 overexpressed. Moreover, NF-κB activation was markedly reversed when HCC cells were treatment with H_2_O_2_ or NAC (an ROS scavenger) in HCC cells (Fig. 5f, g), indicating that CRIF1 activated ROS/NF-κB signaling in HCC cells.

**Fig. 6:**
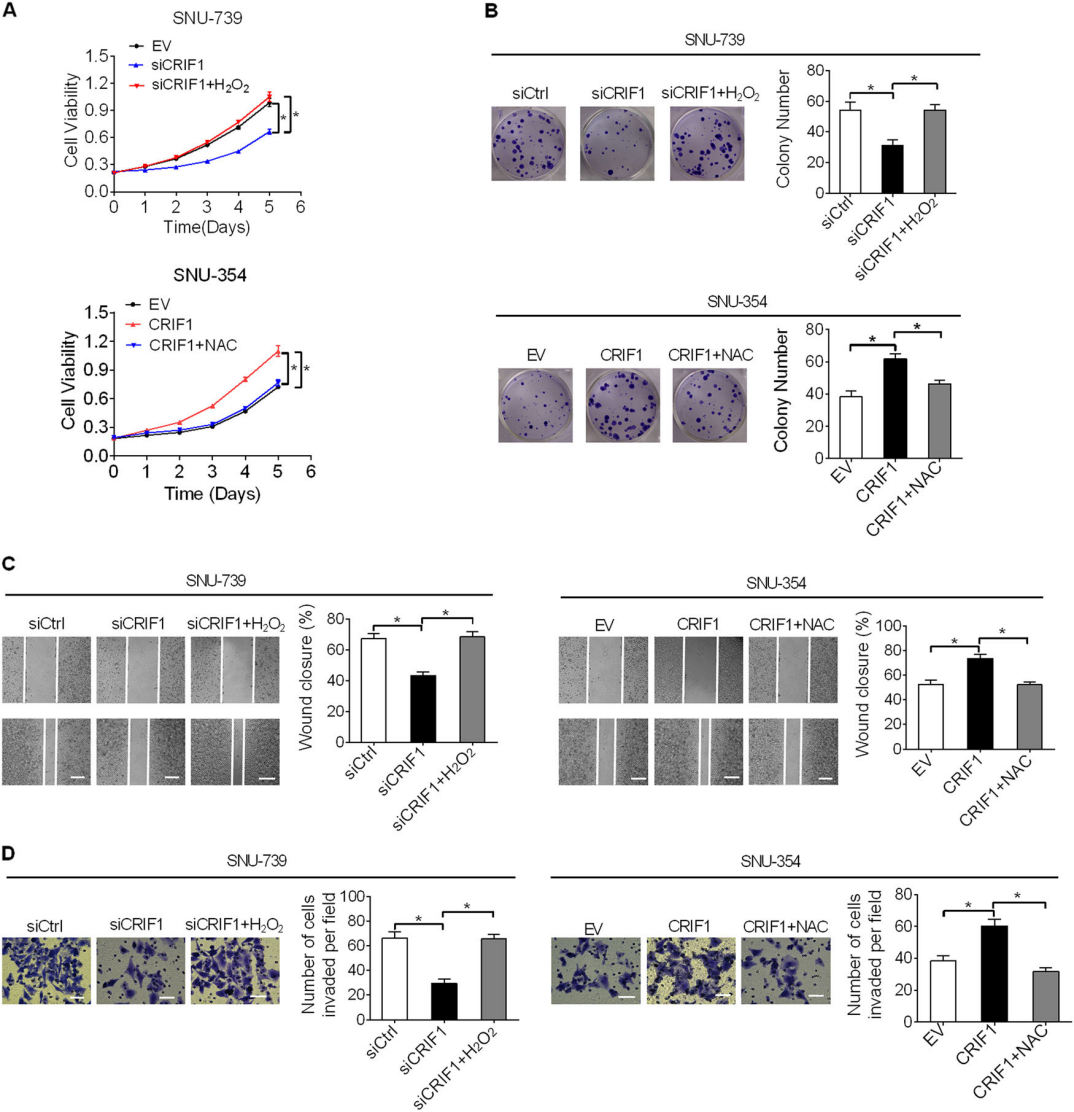
CRIF1-promoted HCC growth and metastasis through activation of ROS/NFκB signaling. **a**, **b** MTS cell viability and colony formation assays in SNU-739 and SNU-354 cells with treatment as indicated. Cells were treated with 100 mM H_2_O_2_ or 20 mM NAC for 12 h. **c**, **d** Wound healing and matrigel invasion assays in SNU-739 and SNU-354 cells with treatment as indicated. Cells were treated with 100 mM H_2_O_2_ or 20 mM NAC for 12 h. Data are expressed as mean ± SEM from three independent experiments. *p < 0.05.

Furthermore, we found that H_2_O_2_ treatment significantly increased both the proliferation and clonogenicity of SNU-739 cells suppressed by CRIF1 knockdown, while NAC treatment attenuated the proliferation and clonogenicity of SNU-354 cells induced by forced CRIF1 expression (Fig. 6a, b). As expected, H_2_O_2_ treatment also significantly promoted the migration and invasion abilities of SNU-739 cells suppressed by CRIF1 knockdown, while NAC treatment inhibited the metastasis of SNU-354 cells induced by forced CRIF1 expression (Figs. 6c, d and S3). Moreover, NF-κB inhibition by BAY-117082 remarkably attenuated CRIF1 overexpression-promoted growth and metastasis of SNU-354 cells (Fig. S2a–d). These results collectively indicated that the oncogenic property of CRIF1 was mediated by the activation of ROS/NFκB signaling.

### Overexpression of CRIF1 was mainly mediated by downregulation of miR-497-5p

**Fig. 7:**
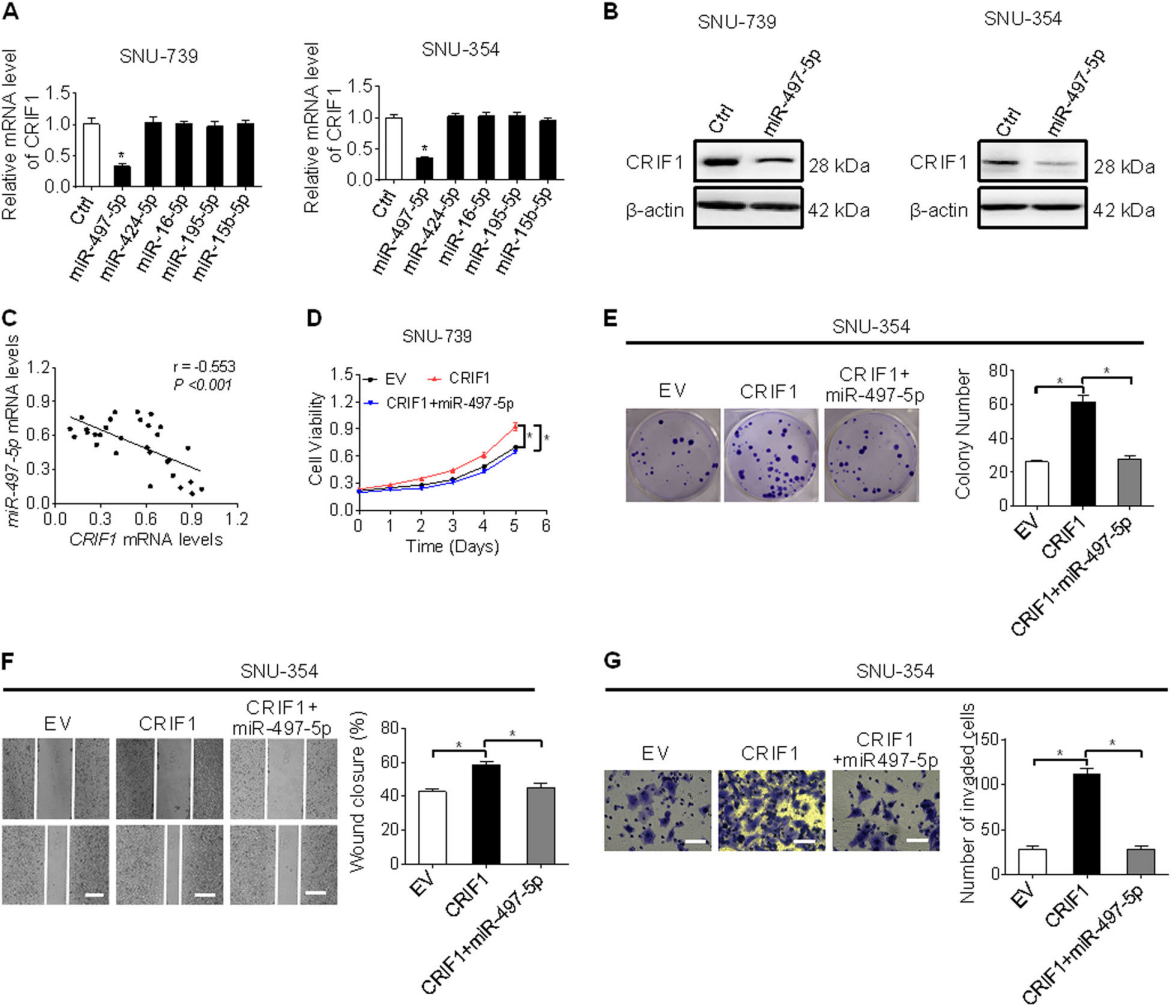
Overexpression of CRIF1 was mainly mediated by downregulation of miR-497-5p. **a** qRT-PCR analysis for CRIF1 expression in SNU-739 cells treated with the top five predicted miRNAs targeting CRIF1. **b** Western blot analysis for CRIF1 expression in SNU-739 and SNU-354 cells after transfection with the synthetic miR-497-5p precursor. **c** Correlation between the mRNA levels of CRIF1 and miR-497-5p was investigated based on the results from qRT-PCR analysis in tumor tissues from 30 HCC patients. **d**, **e** MTS cell viability and colony formation assays in SNU-354 with treatment as indicated. **f**, **g** Wound healing (scale bars, 50 μm) and matrigel invasion (scale bars, 20 μm) assays in SNU-354 cells with treatment as indicated. Data are expressed as mean ± SEM from three independent experiments. *p < 0.05.

MicroRNA (miRNA) plays important roles in the regulation of gene expression. To identify potential miRNA involved in the overexpression of CRIF1 in HCC, miRNA data integration portal-based target prediction^19^ was applied. Among the top five predicted miRNAs targeting CRIF1 (Table S2), only miR-497-5p was demonstrated to repress CRIF1 expression in SNU-739 and SNU-354 cells (Fig. 7a, b). In addition, a significant negative correlation between the levels of miR-497-5p and CRIF1 was found in tumor tissues from 30 HCC patients (Fig. 7c). Moreover, miR-497-5p greatly attenuated the abilities of CRIF1 to promote the growth and metastasis in SNU-354 cells (Fig. 7d–g). Collectively, these results suggest that miR-497-5p represses the expression of CRIF1 and its oncogenic function in HCC cells.

## Discussion

In this study, we show for the first time that CRIF1 was frequently upregulated in HCC cell lines and primary tissues. High expression of CRIF1 was associated with poor OS and RFS in patients with HCC. The clinical outcome of HCC patients varies greatly depending on aggressiveness of the tumors. Although TNM stage has become the most popularly prognostic indicator of disease outcome, a part of HCC patients at the early stages of TNM still have poor prognosis. Accordingly, additional prognostic biomarkers are requested for better risk assessment. Our results indicate that upregulated CRIF1 could be further investigated as a potential prognostic biomarker in HCC.

Upregulation of CRIF1 in HCC cells suggests that CRIF1 may play a role in the progression of HCC. We therefore explored the biological functions of CRIF1 in HCC cells both in vitro and in vivo. Our data showed that knockdown of CRIF1 significantly attenuated cell growth in SNU-739 cells. Conversely, CRIF1 overexpression in SNU-354 cells significantly promoted cell growth. Furthermore, in vivo subcutaneous xenograft models confirmed that CRIF1 significantly increased tumorigenicity of HCC cells in nude mice. In keeping with this, significantly fewer proliferating and more apoptotic cells were detected in CRIF1-knockdown subcutaneous xenograft tumors compared with controls, suggesting that CRIF1 may increase HCC cell growth through inducing G1–S cell cycle transition and inhibiting cell apoptosis/necrosis. However, a previous study in leukemia cells has reported a promotive role for CRIF1 in cell cycle arrest^20^. Possible explanations for this difference include different cellular origins and contexts. In addition, the effects of CRIF1 on HCC cell migration and invasion were also evaluated. Knockdown of CRIF1 significantly inhibited both the migration and invasion abilities of SNU-739 cells, while CRIF1 overexpression markedly increased the migration and invasion abilities of SNU-354 cells.

MiRNA is one of the main regulatory mechanisms of gene expression at the posttranscriptional level. MiR-497-5p is a highly conserved miRNA that has been reported to be downregulated in many different types of cancer^21–24^, including HCC^25^. Here, we show that CRIF1 is a novel target of miR-497-5p in HCC cells. In addition, we found a significant negative correlation between the expression levels of miR-497-5p and CRIF1 in tumor tissues of HCC. These results imply that downregulation of miR-497-5p might be involved in the overexpression of CRIF1 in HCC. However, we still cannot rule out the possibility that other genetic and epigenetic factors could also involved in the upregulation of CRIF1 in HCC cells, which still needs further confirmation.

CRIF1 is a member of mitochondrial ribosome, which is essential for the synthesis of OXPHOS polypeptides in mitochondrial membrane^10^. Several previous studies have reported that CRIF1 was involved in the dysfunction of mitochondrial and thus ROS production^14^. Elevated intracellular ROS level plays critical roles in the promotion of tumor growth and metastasis by activating a series of oncogenic signaling pathways. Besides, several previous studies also have linked increased intracellular superoxide level to cell survival and apoptosis resistance^26–29^. Consistently, our study demonstrated a promotive role for CRIF1 in ROS production in HCC cells. In addition, we demonstrated that elevated ROS production was involved in the promotion of HCC growth and metastasis by CRIF1.

In summary, we found that CRIF1 is frequently overexpressed in HCC and plays an oncogenic role in the progression of HCC by facilitating both tumor growth and metastasis. CRIF1 may serve as a potential prognostic factor and a therapeutic target in HCC.

## Materials and methods

### Reagents

BAY11-7082 was purchased from Calbiochem (San Diego, CA, USA). Commercial kits for detection of SOD, CAT, and GPX activities were purchased from Nanjing Jiancheng Bioengineering Institute (Nanjing, China).

### HCC cell lines and tissue samples collection

All cell lines used in the study including five human HCC cell lines (SNU-354, SNU-368, SNU-739, HLE, and HLF) and one normal human hepatic cell line HL-7702 were obtained from the Cell Bank of the Chinese Academy of Sciences in Shanghai, China. Cells were cultured in medium supplemented with 10% fetal bovine serum (Hyclone) and were authenticated using short-tandem-repeat DNA by the DNA Typing Center in Xi’an, china. In addition, a total of 213 primary HCC tumor tissue samples and paired peritumor tissues (30 with lager size was used for qRT-PCR and western blot analyses and the rest 183 was used for IHC analysis) were collected at the First Affiliated Hospital of Xi’an Jiaotong University in Xi’an, China. All tumor and peritumor tissue samples were histologically confirmed. This study was approved by the Ethics Committee of the First Affiliated Hospital of Xi’an Jiaotong University and written informed consent was obtained from all participants for obtaining the study specimens.

### Knockdown and forced expression of target genes

HCC cells were transfected with siRNA targeting CRIF1 or expression vector of CRIF1 using lipofectamine 2000 (Life Technologies) following to the manufacturer’s instructions. Synthetic miR-497-5p precursor (miR-497-5p) and miR-497-5p inhibitor (anti-miR-497-5p) were used to change the level of miR-497-5p. For shRNA expression vectors targeting CRIF1, a pSilencer™ 3.1-H1 puro vector (Ambion) was used. For overexpression of CRIF1 in HCC cells, the coding sequences of CRIF1 were amplified with primers (Forward: 5′-ATAGGATCCACCATGGCGGCGTCCGTGCGA-3′ and Reverse: 5′-CGCCTCGAGCCTC AGGAGCTGGGTGCCC-3′) from cDNA derived from SNU-739 cells and cloned into the pcDNA^TM^3.1(C) vector (Invitrogen, V790-20).

### RNA extraction and real-time PCR analyses

Trizol reagent (Invitrogen) was used for extraction of total RNA from cell lines or tissue samples of HCC. Extracted RNA was reverse-transcribed into double-stranded cDNA with random primers. PCR reaction was performed using a SYBR Green PCR kit (TAKARA) on a Corbett 6200. Primer sequences were listed in the Table S3.

### Western blot analysis

RIPA buffer was used for lysate preparation and the protein concentration was measured with the BCA protein assay kit (Bio-Rad, Hercules, CA). An amount of 40 µg protein was electrophoresed in SDS-polyacrylamide gels and was then transferred to polyvinylidene fluoride membranes. Membranes were blocked with 5% milk in TBST and incubated with specific primary antibody overnight at 4 °C followed by secondary antibody at room temperature for 2 h, respectively. Protein bands were detected by enhanced chemiluminescence system (ECL; Amersham Pharmacia Biotech). The primary antibodies used were listed in the Table S4.

### IHC analysis

IHC analysis was performed on paraffin sections using an IHC detection kit (Invitrogen) following to the manufacturer’s directions. Briefly, Formalin-fixed, paraffin-embedded tissue sections were deparaffinized in xylenes, rehydrated in a graded series of alcohols, and blocked by 3% hydrogen peroxide for endogenous peroxidase. Then, sections were treated with hot citrate buffer (pH = 6) under pressure for antigen retrieval and incubated with primary and secondary antibodies. The color was developed using DAB and counterstained with hematoxylin. Images were captured under a Olympus light microscopy.

CRIF1 staining results were scored by multiplying the percentage of positive cells by staining intensity. Briefly, the percentage of positive cells were scored as: 0 (0% positive), 1 (1–25% positive), 2 (25–50% positive), 3 (51–75% positive), and 4 (76–100% positive). The staining intensity was graded as follows: 0 for no staining, 1 for weak staining, 2 for moderate staining, and 3 for strong staining. The final semiquantitative IHC score was calculated by multiplying the intensity points by the percentage points.

### MTS cell viability assay

Cell viability was determined by the MTS assay (Promega, G3581) following to the manufacturer’s directions. Briefly, HCC cells were counted using a Scepter 2.1 cell counter and seeded into a 96-well plate (020096, Xinyou Biotech, Hangzhou, China) at a density of 1000 cells/well. After incubation overnight, 20 μl of the MTS solution along with 100 μl of culture media was added to the wells and incubated at 37 °C for 2 h. Cell viability was determined from the absorbance of 490 nm at individual time points of 0, 1, 2, 3, 4, and 5 day after cell transfection.

### Colony formation assay

HCC cells were counted using a Scepter 2.1 cell counter and seeded into six-well plates at a density of 500 cells/well. Cells were cultured in a 37 °C incubator for about 14 days. Colonies were fixed with ice-cold methanol, stained with 0.5% crystal violet, and photographed and counted.

### Cell apoptosis assay

The Annexin V (FITC-conjugated) apoptosis kit (F-6012, US Everbright Inc) was used for determination of cell apoptosis by flow cytometry after staining according to the manufacturer’s instruction. Briefly, HCC cells were collected by centrifugation and then resuspended in 500 ml binding buffer and 5 ml Annexin 5-FITC and propidium iodide (PI). After incubation in the dark for 20 min at room temperature, the apoptotic cells were analyzed by using flow cytometry (Beckman Coulter, Fullerton, CA, USA).

### Cell cycle analysis

Cell cycle distribution at the different stages was analyzed by flow cytometry. Briefly, HCC cells were collected and washed three times with cold phosphate buffered saline. After fixed in 70% cold ethanol at −20 °C for 12 h, cells were stained with 1 ml staining solution in 50 U/ml RNase and 50 μg/ml PI, and incubated in the dark for 40 min at 4 °C. Finally, cell cycle distribution was analyzed with flow cytometry (Beckman Coulter, Fullerton, CA, USA).

### Wound healing assay

Cell migration was assessed by the scratch wound healing assay. Briefly, HCC cells were counted using a Scepter 2.1 cell counter and seeded into six-well plates. After grown to 90% confluency, a scratch was performed vertically in the middle of the wells with a 200 μl micropipette tip to create an artificial wound zone. Floating and detached cells were removed by washing with serum-free medium. The scratched monolayer of HCC cells was photographed (time 0 h and 24 h) using an inverted light microscope.

### Matrigel invasion assay

Transwell chambers (24-well) with matrix gel were used for cell invasive ability analysis. Briefly, HCC cells were counted using a Scepter 2.1 cell counter and 1 × 10^5^ cells were seeded on to the upper chamber wells and then incubated in serum-free culture medium for 24 h. Non-migrating cells in the upper chamber were removed and penetrated cells on the bottom were fixed with 4% paraformaldehyde and stained with crystal violet (Beyotime, Shanghai, China) and photographed

### Tumor xenograft mouse model

A total of 5 × 10^6^ stable CRIF1-knockdown (shCRIF1) or control (shCtrl) cells were injected subcutaneously into the dorsal right flank of each 6-week-old male Balb/c nude mice randomly (six mice per group). Tumor volumes were measured by a vernier caliper weekly for 5 weeks to calculate the tumor volume. At the endpoint (5 weeks post injection), the mice were sacrificed and tumors were excised and weighed. All experimental procedures were approved by the Institutional Animal Care and Use Committee at Xian Jiaotong University.

### Tail vein metastatic assay

A total of 2 × 10^6^ stable CRIF1-knockdown (shCRIF1) or control (shCtrl) cells were injected intravenously through the tail vein into the 6-week-old male Balb/c nude mice (six mice per group). The mice were sacrificed 8 weeks post injection and the lungs were excised and paraffin embedded. Then, hematoxylin and eosin staining was performed and metastatic tumor nodules formed in the lungs were counted.

### Detection of cellular ROS level and activities of antioxidants

Cellular ROS level was measured by a fluorescent probe DCFH-DA (Beyotime Biotechnology, S0033) following to the manufacturer’s instructions. Briefly, HCC cells were incubated with DCFH-DA at a concentration of 10 mM in serum-free medium at 37 °C for 20 min. Flow cytometry was used for the assessment of the fluorescence in each group.

Activities of antioxidants of SOD, CAT, and GPX were determined with commercial kits from Nanjing Jiancheng Bioengineering Institute (Nanjing, China) following to the manufacturer’s instruction. One-unit (U) activity was defined as the amount of antioxidant enzyme consuming 1 μmol of substrate/min/mg total protein (U/mg prot).

### Statistical analysis

The data are presented as mean ± standard error of the mean (SEM). Statistical analyses were performed using SPSS 17.0 software (SPSS, Chicago, IL). The *χ*2 test was used to compare the clinicopathological characteristics of HCC patients and CRIF1 expression. For compare the difference between two groups, paired or unpaired student’s *t* test was used. Multiple group comparisons were analyzed by one-way ANOVA. Correlations between measured variables were tested by Pearson correlation analyses. Overall and progression free survival in relation to CRIF1 expression were evaluated from Kaplan–Meier survival curves and the log-rank test. *P* value < 0.05 was taken as statistical significance.

## Supplementary information

Supplementary Figure 1

Supplementary Figure 2

Supplementary Figure 3

supplementary figures and tables
