# Supplementary figures and images for: CRIF1 overexpression facilitates tumor growth and metastasis through inducing ROS/NFκB pathway in hepatocellular carcinoma
Source: Cell Death Dis. 2020 May 7;11(5):332. doi: 10.1038/s41419-020-2528-7 (PMC7205899; doi:10.1038/s41419-020-2528-7)

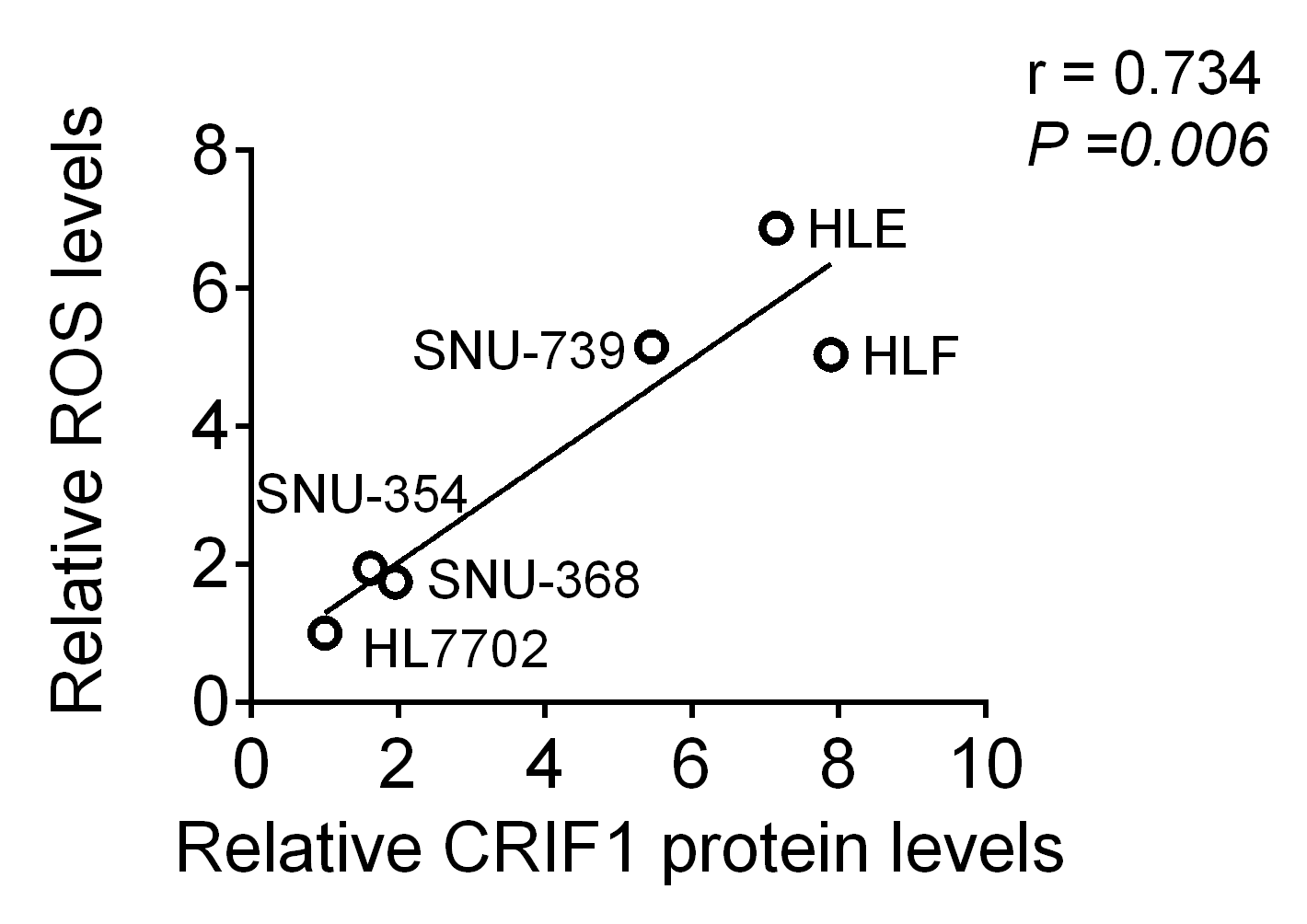

Supplement: Supplementary file 1 — Supplementary Figure 1 [file 41419_2020_2528_MOESM1_ESM.tif]

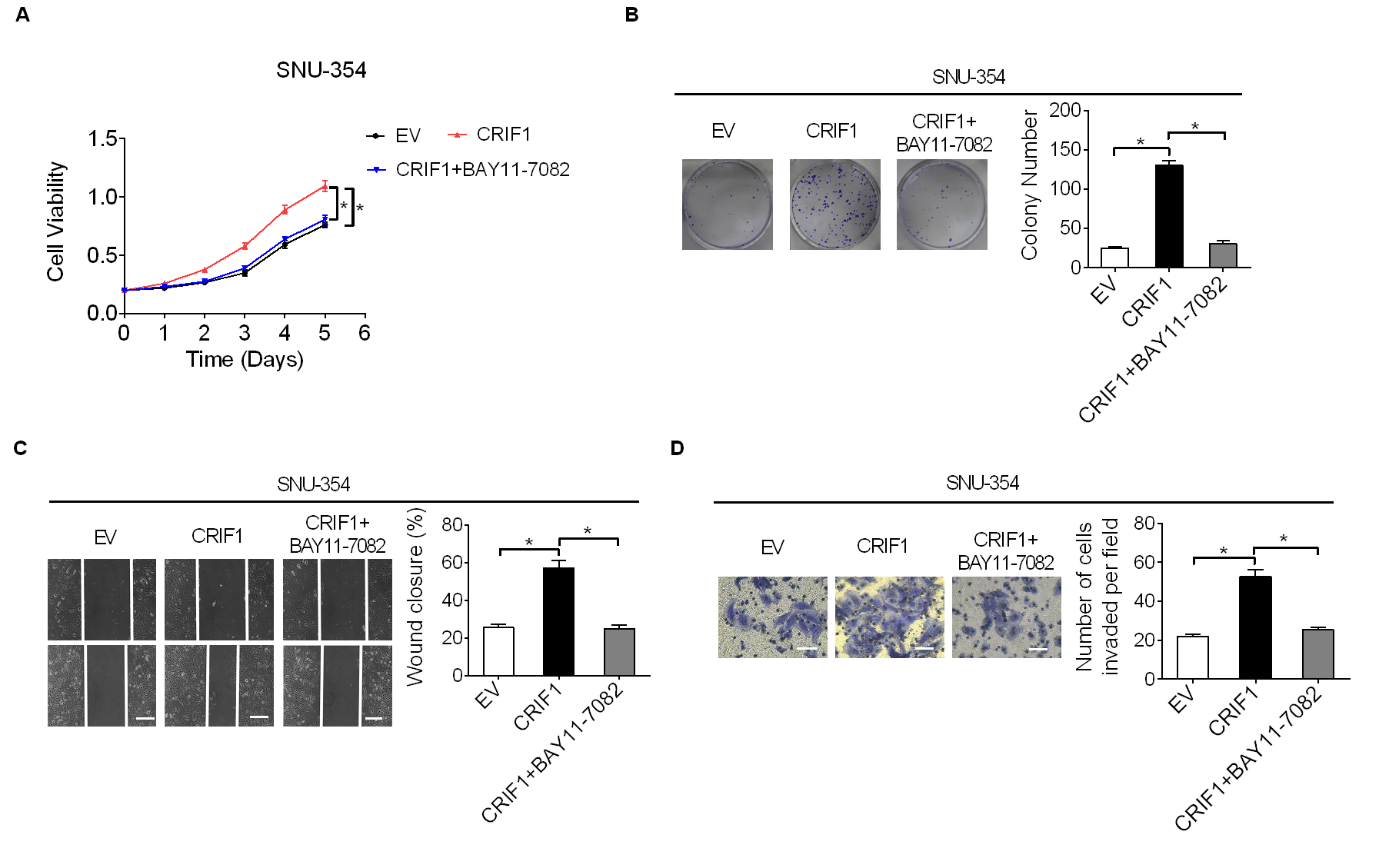

Supplement: Supplementary file 2 — Supplementary Figure 2 [file 41419_2020_2528_MOESM2_ESM.tif]

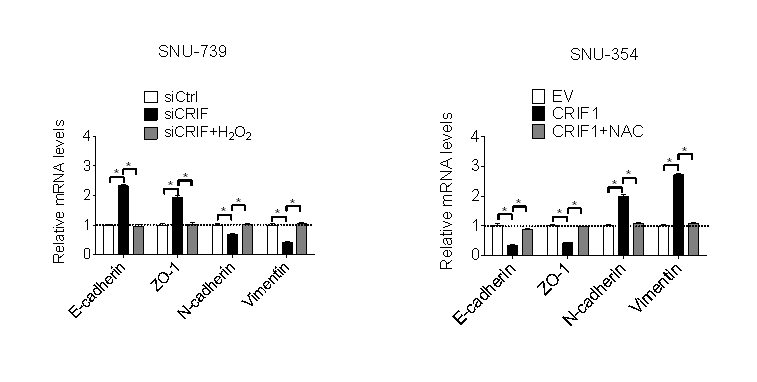

Supplement: Supplementary file 3 — Supplementary Figure 3 [file 41419_2020_2528_MOESM3_ESM.tif]
