# Supplementary material for: CRIF1 overexpression facilitates tumor growth and metastasis through inducing ROS/NFκB pathway in hepatocellular carcinoma
Source: Cell Death Dis. 2020 May 7;11(5):332. doi: 10.1038/s41419-020-2528-7 (PMC7205899; doi:10.1038/s41419-020-2528-7)
Supplement: Supplementary file 4 — supplementary figures and tables [file 41419_2020_2528_MOESM4_ESM.doc]

**CRIF1 over-expression facilitates tumor growth and metastasis through inducing ROS/ NFκB pathway in hepatocellular carcinoma、**

**Supplementary Figure 1.** Correlation between protein expression levels of CRIF1 and ROS levels in five human HCC and one normal hepatic cell lines.

**Supplementary Figure 2. (A and B)** MTS cell viability and colony formation assays in SNU-354 with treatment as indicated. Cells were treated with Bay11-7082 (12.5 mM) for 12 h. **(C and D)** Wound healing (Scale bars, 50 μm) and matrigel invasion (Scale bars, 20 μm) assays in SNU-354 cells with treatment as indicated. Cells were treated with Bay11-7082 (12.5 mM) for 12 h. Data are expressed as mean ± SEM from three independent experiments. *p < 0.05.

**Supplementary Figure 3.** Quantitative RT-PCR analysis for expression levels of EMT markers (E-cadherin, ZO-1, N-cadherin and Vimentin) in SNU-739 and SNU-354 cells with indicated treatment. Cells were treated with 100 mM H_2_O_2_ or 20 mM NAC for 12 h. Data are expressed as mean ± SEM from three independent experiments. *p < 0.05.

**Supplementary Table 1.** Association between CRIF1 expression and clinicopathologic features in 183 patients with HCC.

| Variables | No. of cases (%) | CRIF1 expression | | *P* value |
| --- | --- | --- | --- | --- |
|  |  | Low | High |  |
| All | 183 (100%) | 90 | 93 |  |
| Age |  |  |  |  |
| <55 | 81 (44.3%) | 39 | 42 | 0.882 |
| >=55 | 102 (55.7%) | 51 | 51 |  |
| Gender |  |  |  |  |
| Female | 31 (16.9%) | 17 | 14 | 0.557 |
| Male | 152 (83.1%) | 73 | 79 |  |
| HBsAg |  |  |  |  |
| Negative | 19 (10.4%) | 11 | 8 | 0.474 |
| Positive | 164 (89.6%) | 79 | 85 |  |
| AFP (ug/ml) |  |  |  |  |
| <200 | 99 (54.1%) | 47 | 52 | 0.658 |
| >=200 | 84 (45.9%) | 43 | 41 |  |
| Maximum diameter of lesion |  |  |  |  |
| <5 | 148 (80.9%) | 79 | 69 | **0.024** |
| >=5 | 35 (19.1%) | 11 | 24 |  |
| PVTT |  |  |  |  |
| No | 143 (78.1%) | 69 | 74 | 0.721 |
| Yes | 40 (21.9%) | 21 | 19 |  |
| TNM stage |  |  |  |  |
| I+ II | 132 (72.1%) | 73 | 59 | **0.009** |
| III+ IV | 51 (27.9%) | 17 | 34 |  |
| Differentiation grade |  |  |  |  |
| I+ II | 60 (32.8%) | 28 | 32 | 0.641 |
| III | 123 (67.2%) | 62 | 61 |  |
| Treatment |  |  |  |  |
| Hepatectomy | 129 (70.5%) | 67 | 62 | 0.261 |
| Hepatectomy+ TACE | 54 (29.5%) | 23 | 31 |  |

**Abbreviations**: AFP, alpha-fetoprotein; HBsAg, hepatitis B virus surface antigen; PVTT, portal vein tumor thrombosis; TACE, transcatheter arterial chemoembolization. TNM, tumor-nodes-metastases;

**Supplemental table 2. The top five predicted miRNAs targeting CRIF1 using microRNA Data Integration Portal (mirDIP).**

**
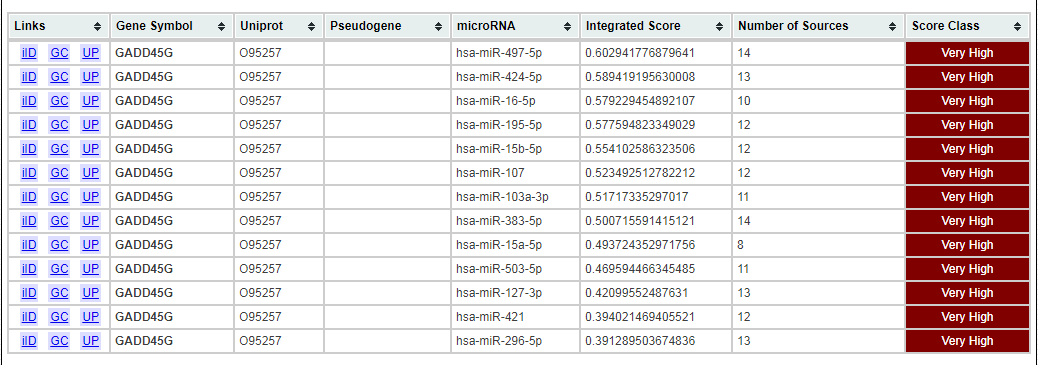
**

**Supplementary Table 3. Sequence of primers for qRT-PCR analysis**

| **1. Primers used in q-PCR analysis** | | |  |
| --- | --- | --- | --- |
| *CRIF1* | forward primer | GGCCCAGGCTGACAAGGAGAG | |
|  | reverse primer | GCGCCTCCTTCTTCCGTTTCTGT | |
| *E-cadherin* | forward primer | AAAGGCCCATTTCCTAAAAACCT | |
|  | reverse primer | TGCGTTCTCTATCCAGAGGCT | |
| *Z0-1* | forward primer | CGACCAGATCCTCAGGGTAA | |
|  | reverse primer | TCCATAGGGAGATTCCTTCTCA | |
| *N-cadherin* | forward primer | TCAGGCTGTGGACATAGAAACC | |
|  | reverse primer | GCTGTAAACGACTCTGGCACT | |
| *Vimentin* | forward primer | GACGCCATCAACACCGAGTT | |
|  | reverse primer | CTTTGTCGTTGGTTAGCTGGT | |
| *β-actin* | forward primer | ACCCCGTGCTGCTGACCGAG | |
|  | reverse primer | TCCCGGCCAGCCAGGTCCA | |
| *miR-479-5p* | forward primer | ACACTCCAGCTGGGAGTGGGGAACCCTTC | |
|  | reverse primer | TGGTGTCGTGGAGTCG | |
| *U6* | forward primer | CTCGCTTCGGCAGCACA | |
|  | reverse primer | AACGCTTCACGAATTTGCGT | |

**Supplementary Table 4.** Primary antibodies used for western blot and immunohistochemistry.

| **Antibody** | **Company (Cat. No.)** | **Working dilutions** |
| --- | --- | --- |
| CRIF1 | abcam (ab244530) | WB: 1/1000 IHC: 1/200 |
| E-cadherin | abcam (ab1416) | WB: 1/1000 |
| ZO-1 | abcam (ab190085) | WB: 1/1000 |
| N-cadherin | abcam (ab98952) | WB: 1/1000 |
| Vimentin | abcam (ab8978) | WB: 1/1000 |
| ki-67 | abcam (ab15580) | IHC: 1/500 |
| Akt | Proteintech (10176-2-AP) | WB: 1/1000 |
| p-Akt (Ser473) | Proteintech (66444-1-IG) | WB: 1/1000 |
| p65 | Proteintech (10745-1-AP) | WB: 1/1000 |
| p-p65 (Ser536) | Abcam (b86299) | WB: 1/800 |
| Hif1α | Abcam (ab92498) | WB: 1/600 |
| Lamin B1 | Proteintech (12987-1-AP) | WB: 1/800 |
| β-actin | Proteintech (20536-1-AP) | WB: 1/1000 |
